# A Much-Needed Home for Open-Air Treatment

**Published:** 1903-05-16

**Authors:** 


### EDITOR'S LETTER-BOX.

**[Our Correspondents are reminded that prolixity is a great bar to publication, and that brevity of style and conciseness of statement greatly facilitate early insertion.]**

### A MUCH-NEEDED HOME FOR OPEN-AIR TREATMENT.

DR. EDWARD DEANESLY, F.R.C.S., hon. surgeon to the General Hospital, Wolverhampton, writes: "Some of your readers may be glad to hear of a medical home where patients who are unable to obtain admission to large and expensive sanatoria may obtain the advantages of open-air treatment and suitable climate at a moderate cost. Such a home is provided for a limited number of not too acute or severe cases of tuberculosis, anæmia or convalescence from illness by Mrs. Mary White, of Penmaenmawr. Mrs. White was formerly matron of the General Hospital, Wolverhampton, and has since been matron of a well-known sanatorium for consumption. A letter, which I received from the relative of a patient whom I recommended to her, speaks very enthusiastically of the benefits received. Perhaps this information may be of use to others similarly situated."

[Details of this Home will be found in the Nursing Section, page 85. All communications should be addressed to Mrs. Mary White, Bronwylfa, Penmaenmawr.—ED. THE HOSPITAL.]

